# Renal tubular epithelial cells: the neglected mediator of tubulointerstitial fibrosis after injury

**DOI:** 10.1038/s41419-018-1157-x

**Published:** 2018-11-13

**Authors:** Ruochen Qi, Cheng Yang

**Affiliations:** 1 Department of Urology, Zhongshan Hospital, Fudan University, 200032 Shanghai, P. R. China; 2 Shanghai Medical College, Fudan University, 200032 Shanghai, P.R. China; 3 Shanghai Key Laboratory of Organ Transplantation, 200032 Shanghai, P. R. China

## Abstract

Renal fibrosis, especially tubulointerstitial fibrosis, is the inevitable outcome of all progressive chronic kidney diseases (CKDs) and exerts a great health burden worldwide. For a long time, interests in renal fibrosis have been concentrated on fibroblasts and myofibroblasts. However, in recent years, growing numbers of studies have focused on the role of tubular epithelial cells (TECs). TECs, rather than a victim or bystander, are probably a neglected mediator in renal fibrosis, responding to a variety of injuries. The maladaptive repair mechanisms of TECs may be the key point in this process. In this review, we will focus on the role of TECs in tubulointerstitial fibrosis. We will follow the fate of a tubular cell and depict the intracellular changes after injury. We will then discuss how the repair mechanism of tubular cells becomes maladaptive, and we will finally discuss the intercellular crosstalk in the interstitium that ultimately proceeds tubulointerstitial fibrosis.

## Facts

- TECs are vulnerable to injury in a variety of CKDs.
- TECs are actually participants, rather than victims or bystanders, in the progression of renal fibrosis.Please check your article carefully, coordinate with any co-authors and enter all final edits clearly in the eproof, remembering to save frequently. Once corrections are submitted, we cannot routinely make further changes to the article.Article checked
- TECs can start repair mechanisms upon injury, though these mechanisms sometimes aggravate renal fibrosis.
- TECs interact with other cell types in the interstitium, leading to renal fibrosis.

Note that the eproof should be amended in only one browser window at any one time; otherwise changes will be overwritten.Understood, thanks for reminding.

## Open questions

- Are TECs profibrotic or antifibrotic in the progression of tubulointerstitial fibrosis?Author surnames have been highlighted. Please check these carefully and adjust if the first name or surname is marked up incorrectly. Note that changes here will affect indexing of your article in public repositories such as PubMed. Also, carefully check the spelling and numbering of all author names and affiliations, and the corresponding email address(es).Authors' names have been checked. They are correct.
- How do TECs change the microenvironment in the interstitium upon injury?
- Where is the borderline between beneficial repair and maladaptive repair for TECs upon injury?
- Are cell senescence and epithelial–mesenchymal transition different facets of the same process?
- Is it possible to target TECs to alleviate CKDs in clinical settings in the future?

## Introduction

Chronic kidney diseases (CKDs) have exerted a great burden on public health worldwide. According to the WHO estimate, CKD accounted for 1.5% of deaths worldwide in 2012^1^. Renal fibrosis, especially tubulointerstitial fibrosis (TIF), is the inevitable outcome of all progressive CKD^2^, and therefore, exploring the intrinsic mechanisms of TIF is of great importance. TIF is manifested by tubular atrophy and the accumulation of extracellular matrix (ECM)^3^. For a long time, interests have focused on fibroblasts and myofibroblasts. However, in recent years, a growing number of studies are shedding light on the role of tubular epithelial cells (TECs) in renal fibrosis^4^. Evidence has shown that TECs, rather than being victims or bystanders, are probably an initiator of the TIF response to a variety of injuries^5^. The maladaptive repair mechanisms of TECs can be the key point of progression from acute to chronic disease^6^. In this review, we will focus on the role of TECs as an important mediator of TIF upon injury.Please note that after the paper has been formally accepted you can only provide amended Supplementary Information files for critical changes to the scientific content, not for style. You should clearly explain what changes have been made if you do resupply any such files.Understood. Thanks for reminding.Should you wish to order offprints, please click on www.nature.com/aj/forms/bmt_offprint_2017.pdf to download and complete the offprint form and upload the completed form along with the article.Understood. Thanks for reminding.

## What are the intracellular changes of TECs upon injury?

### Mitochondria dysfunction and reactive oxygen species (ROS) aggravate tubular injury

**Fig. 1:**
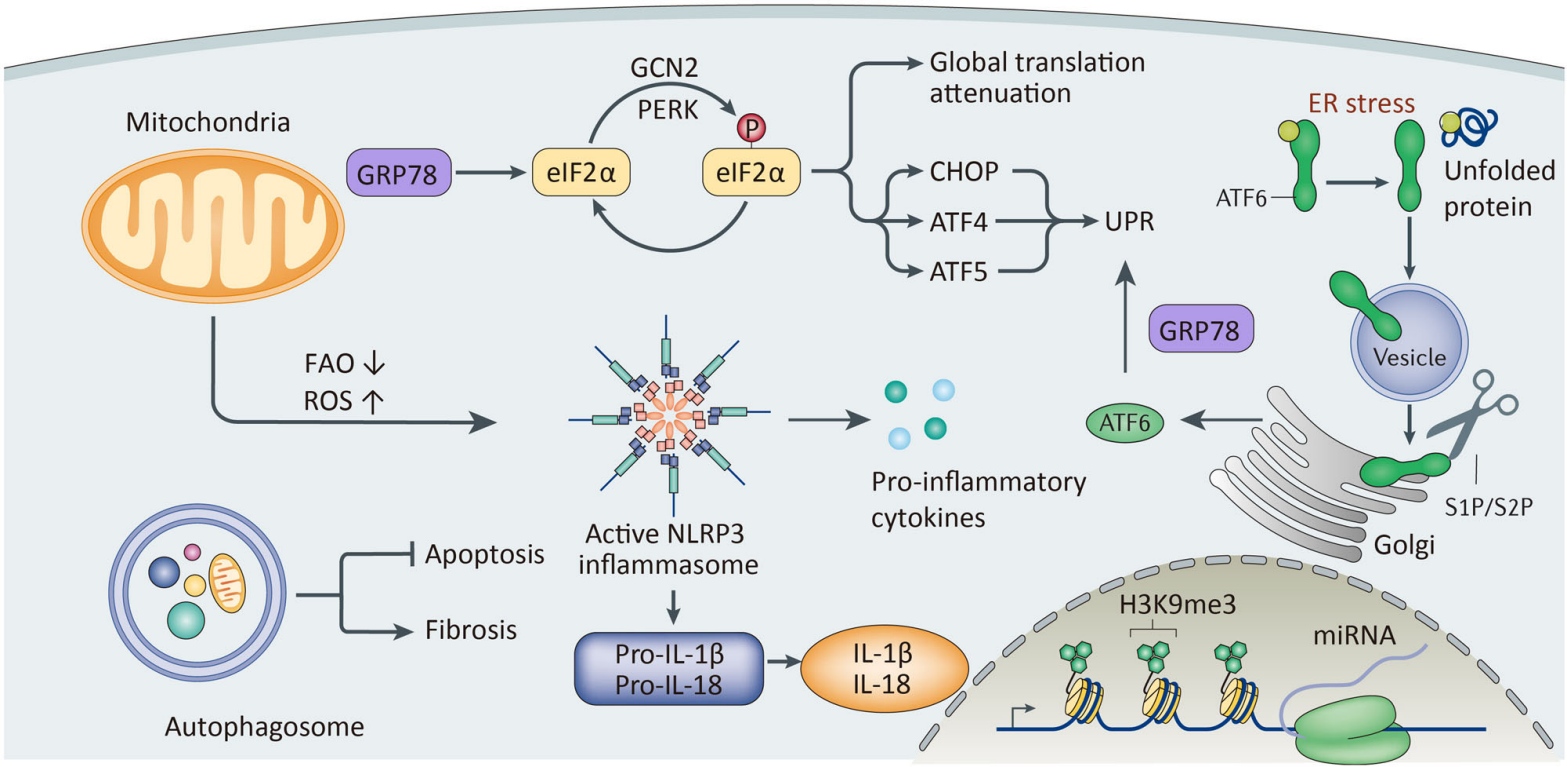
A schematic view of intracellular changes in tubular cells upon injury. Mitochondria undergo metabolic disorders, manifested by decreased fatty acid oxidation. Mitochondria can also produce ROS and further activate the NLRP3/inflammasome. Production of mitochondrial ROS is also related to the expression of proinflammatory factors. ER stress caused by misfolded proteins can start the unfolded protein reaction (UPR), including activation of GRP78 and downstream signaling, including the PERK–eIF2–ATF4 pathway and ATF6 pathway. However, persistent activation of UPR can result in apoptosis. Autophagy plays a dual role. This process can either avoid apoptosis or aggravate renal fibrosis, depending on different situations. Injuries can also lead to epigenetic changes and changes in mRNA expression

Mitochondrial impairment may aggravate TEC injury by disturbing energy metabolism and activating ROS and NLR family pyrin domain containing 3 (NLRP3)/inflammasomes (Fig. 1). A genome-wide transcriptome study on kidney biopsy specimens with TIF showed the deposition of lipid in TECs along with decreased expression of enzymes related to fatty acid oxidation (FAO)^7^. Inhibition of FAO leads to a fibrotic phenotype change in TECs, while restoring FAO attenuates TIF^7^. The impairment of FAO is attributed to the downregulation of peroxisome proliferator-activated receptor α  and overexpression of miR-21^8–10^. The loss of FAO also drives a metabolic switch to glycolysis in TECs to meet energy demands^11^. Impaired mitochondrial function also leads to the production of ROS and oxidative stress in TECs^8, 12^. Studies on diabetic nephropathy (DN) show that the accumulation of advanced oxidation protein products (AOPPs) results in mitochondrial injury and oxidative stress^12^. Production of ROS in the mitochondria can further lead to the activation of NLRP3^13–15^. Two earlier studies showed increased mitochondrial ROS production along with NLRP3/inflammasome activation. Inhibition of mitochondrial ROS production simultaneously inhibits the activation of NLRP3 and downstream interleukin-1β (IL-1β) and IL-18 production, indicating the presence of mitochondrial ROS prior to the activation of NLRP3^13, 14^^,^. A recent study showed that this process is probably mediated by the mROS–TXNIP–NLRP3 pathway^15^. Mitochondrial ROS can also increase the expression of several other proinflammatory and profibrotic factors, including monocyte chemoattractant protein-1 (MCP-1), plasminogen activator inhibitor and transforming growth factor beta 1 (TGF-β1)^16^.

Please define NLRP3 at its first mention in text.NLRP3 stands for NLR family pyrin domain containing 3Please spell out PPARα in text, as it is mentioned only once.PPARalpha stands for peroxisome proliferator-activated receptor α

These results indicate that improving normal mitochondrial function may attenuate renal fibrosis. Recent studies prove the potential renoprotective role of several intrinsic modulation factors that are closely related to mitochondrial function. Either downregulation of dynamin-related protein 1 ^17^ or upregulation of NAD-dependent deacetylase sirtuin-3, mitochondrial ^18^ or TNF receptor associated protein 1 ^19^ could have a protective effect on mitochondrial function in TECs and ameliorate renal fibrosis.Please spell out DRP117, SIRT318 and TRAP1in text, as it is mentioned only once.DRP1 stands for dynamin-related protein 1SIRT3 stands for NAD-dependent deacetylase sirtuin-3, mitochondrialTRAP1 stands for TNF receptor associated protein 1 (TRAP1)

### Endoplasmic reticulum (ER) stress and unfolded protein response (URP) are double-edge swords in tubular injury

Injuries by toxins, albuminuria, and metabolic disorders lead to accumulation of misfolded proteins in TECs and ER stress. ER stress is manifested by UPR^20^, an intrinsic adaptive process that helps cells survive. However, persistent activation of UPR may finally lead to apoptosis^21^.

GRP78, a molecular chaperone that functions in protein folding, makes UPR a pro-survival mechanism (Fig. 1). In an age-related proteinuria model, more severe TIF was observed in the aged group, together with a significantly suppressed GRP78 level, indicating the protective role of GRP78^22^. The binding of misfolded protein to GRP78 can start three pathways, the PRKR-like endoplasmic reticulum kinase-eukaryotic translation initiation factor 2-activating transcription factor 4 (PERK–eIF2–ATF4) pathway, inositol-requiring enzyme 1-X-Box binding protein 1  pathway and activating transcription factor 6  pathway, which in turn can activate UPR and mediate cell repair^23^. A recent study has also found the involvement of the mitogen-activated protein kinase (MAPK/ERK) pathway in ER stress^24^. However, persistent injury and UPR activation can lead to cell apoptosis. This process is mediated by caspase-12, an ER-specific caspase, via activation of the caspase-9/caspase-3 cascade^20^. A recent study has also shed light on the profibrotic role of a novel ER-associated protein, RTN1A^21, 25^. RTN1A is related to prolonged ER stress and apoptosis of TECs^25^. Specific knockout of *RTN1A* in TECs could downregulate ER stress signaling and ameliorate ER stress-associated apoptosis^21^.Please define PERK–eIF2–ATF4, IRE1-XBP1, ATF6, MAPK/ERK at its first mention in text.PERK–eIF2–ATF4 stands for PRKR-like endoplasmic reticulum kinase-eukaryotic translation initiation factor 2-activating transcription factor 4IRE1–XBP1 stands for inositol-requiring enzyme 1-X-Box binding protein 1ATF6 stands for activating transcription factor 6MAPK/ERK stands for mitogen-activated protein kinase

Recognition of these facts has also offered some possible approaches for the diagnosis and treatment of renal fibrosis. Urinary cysteine-rich with EGF-like domains 2, a molecule induced and secreted upon ER stress from podocytes and TECs, could be a prospective predictor of tubulointerstitial injury^26^. The chemical chaperon sodium 4-phenylbutyrate can mimic the role of an ER chaperone in vivo and greatly reduce TECs apoptosis and renal fibrosis caused by ER stress^27^. Another molecule, HMG-CoA reductase degradation 1 homolog , an E3 ubiquitin ligase, can also ameliorate TEC apoptosis by promoting ubiquitylation and degradation of eIF2alpha, a critical protein in the PERK–eIF2alpha–ATF4–CHOP signaling pathway^28^.Please spell out HRD1 in text, as it is mentioned only once.HRD1 stands for HMG-CoA reductase degradation 1 homolog

### Autophagy exerts bidirectional effects on TECs upon injury

Autophagy is a mechanism that engulfs unnecessary or dysfunctional cellular components and can help TECs adapt to a variety of injuries;^29–31^ however, persistent activation of autophagy leads to fibrosis (Fig. 1). In DN, the specific inhibition of autophagy results in the accumulation of advanced glycation end products along with worsened inflammation and fibrosis^29^. Two other studies focusing on DN also found an association between decreased autophagy and renal fibrosis, and this process is probably the result of inhibition of phosphatase and tensin homolog  by Notch1/Hes1 in response to high glucose concentrations^32, 33^. Another in vitro study showed that enhanced autophagy ameliorates fibrosis induced by TGF-β1^30^. Increased autophagy is observed along with decreased apoptosis^31^. One study has also shown that autophagy is partly regulated by ER stress to improve renal function and histology^34^.Please spell out PTEN in text, as it is mentioned only once.PTEN stands for phosphatase and tensin homolog

Two studies focusing on the role of Atg5 had controversial results^35, 36^. One showed that genetic deletion of Atg5-mediated autophagy specifically in TECs leads to marked cell cycle arrest, increased COL1 deposition, and severe interstitial fibrosis in an unilateral ureteral obstruction (UUO) model^37^. However, the other study on Atg5 indicated that the ablation of autophagy in TECs leads to significantly less tubular senescence and reduced interstitial fibrosis 30 days after ischemia–reperfusion injury^35^. Moreover, another study investigating kidney proximal tubule-specific *Atg7* knockout mice also showed ameliorated tubular injury and TIF^36^. These results indicate the two-way effect of autophagy in renal fibrosis. Indeed, autophagy can degrade unnecessary or dysfunction components and prevent cell apoptosis. However, some severely damaged TECs that should have undergone apoptosis may also manage to survive via autophagy. These cells undergo maladaptive repair and phenotype changes, thereby becoming sources of proinflammatory and profibrotic cytokines, leading to aggravated renal fibrosis^35^.Please define UUO at its first mention in text.UUO stands for unilateral ureteral obstruction

### Epigenetic changes in TECs provide new therapeutic insights

One study on DN revealed a switch in DNA methylation and changes of mRNA expression of select genes, thereby leading to phenotype changes of TECs^38^. Another study showed increased H3K9 histone markers after UUO, indicating that epigenetics may play a role in fibrogenesis^39^. The possible involvement of epigenetics provides a new aspect of treatment for renal fibrosis (Fig. 1). Two research groups using either MS 275 or valproic acid  demonstrated that the class I histone deacetylase  inhibitor could effectively ameliorate renal fibrosis caused by UUO^40, 41^. A recent study applied DZNep (3-deazaneplanocin A), an H3K27me3 inhibitor, in an ischemia–reperfusion model. TECs harvested from the model were examined with high-throughput sequencing (RNA-seq). The study showed that profibrotic genes such as *COL3A1* (collagen type 3a1) and *TIMP2* (tissue inhibitor of metalloproteinase 2) are suppressed by DZNep in vivo^42^, demonstrating the efficacy of the treatment at the RNA level. Epigenetic changes in TECs may be an important research area for investigating the mechanisms of, or the therapeutic approaches for, renal fibrosis.Please spell out MS 275, VPA and HDAC in text, as it is mentioned only once.Both MS 275 and VPA are drugs used as HDAC inhibitor.MS 275 is the name of the drug, not an abbreviation.VPA stands for valproic acid.HDAC stands for histone deacetylase.Per style, gene names have to be italicized. Hence, COL3A1 and TIMP2 have been italicized where mentioned as genes. Please confirm that the italicization is appropriate and also mark if there are further instances that need to be italicized.Genes are italicized, thanks for reminding.

### Important signaling pathways involved in renal fibrosis

A great number of intracellular pathways are involved in TECs upon injury (Table 1), of which the TGF-beta1/Smad3 pathway^43–45^, RAS pathway^46–48^, and Wnt/beta-catenin pathway^49, 50^ are the most well established. In addition to these, several other pathways are also involved, including the p38 MAPK pathway^24, 51, 52^, phosphatidylinositide 3-kinases/protein kinase B  pathway^53–57^, epidermal growth factor receptor (EGFR) pathway^58–60^, Hippo-Salvador pathway^61^, Notch pathway^33, 62^, c-Jun N-terminal kinases (JNK) pathway^63^, Sonic hedgehog pathway^64^, adiponectin/adiponectin receptor 1  pathway^65^, membrane rafts–redox pathway, etc^66^. Each signaling pathway may exert partial influences that drive the cell to play either a profibrotic or antifibrotic role, whereas different pathways can interact with each other at different levels, forming an intricate signaling network that regulates the progression of renal fibrosis after different injuries. The fact that the signaling pathways regulating renal fibrosis are still not well understood urges thorough investigation. A more comprehensive description of the regulation network will provide us with more potential targets for preventing or even reversing renal fibrosis.

**Table 1:** Important signaling pathways involved in renal fibrosis

Signaling pathway	Role in renal fibrosis	Reference
TGF-beta1/Smad3 pathway	Profibrotic	^43– 45^
RAS pathway	Profibrotic and antifibrotic	^46– 48^
Wnt/beta-catenin pathway	Profibrotic and antifibrotic	^49, 50^
P38 MAPK pathway	Profibrotic	^24, 51, 52^
PI3K/AKT pathway	Profibrotic	^53– 57^
EGFR pathway	Profibrotic and antifibrotic	^58– 60^
Hippo-Salvador pathway	Antifibrotic	^61^
Notch pathway	Profibrotic	^33, 62^
JNK pathway	Profibrotic	^63^
Sonic hedgehog pathway	Profibrotic	^64^
APN/AdipoR1 pathway	Profibrotic	^65^
Membrane rafts–redox pathway	Profibrotic	^66^

## How do repair mechanisms become maladaptive and result in renal fibrosis?

**Fig. 2:**
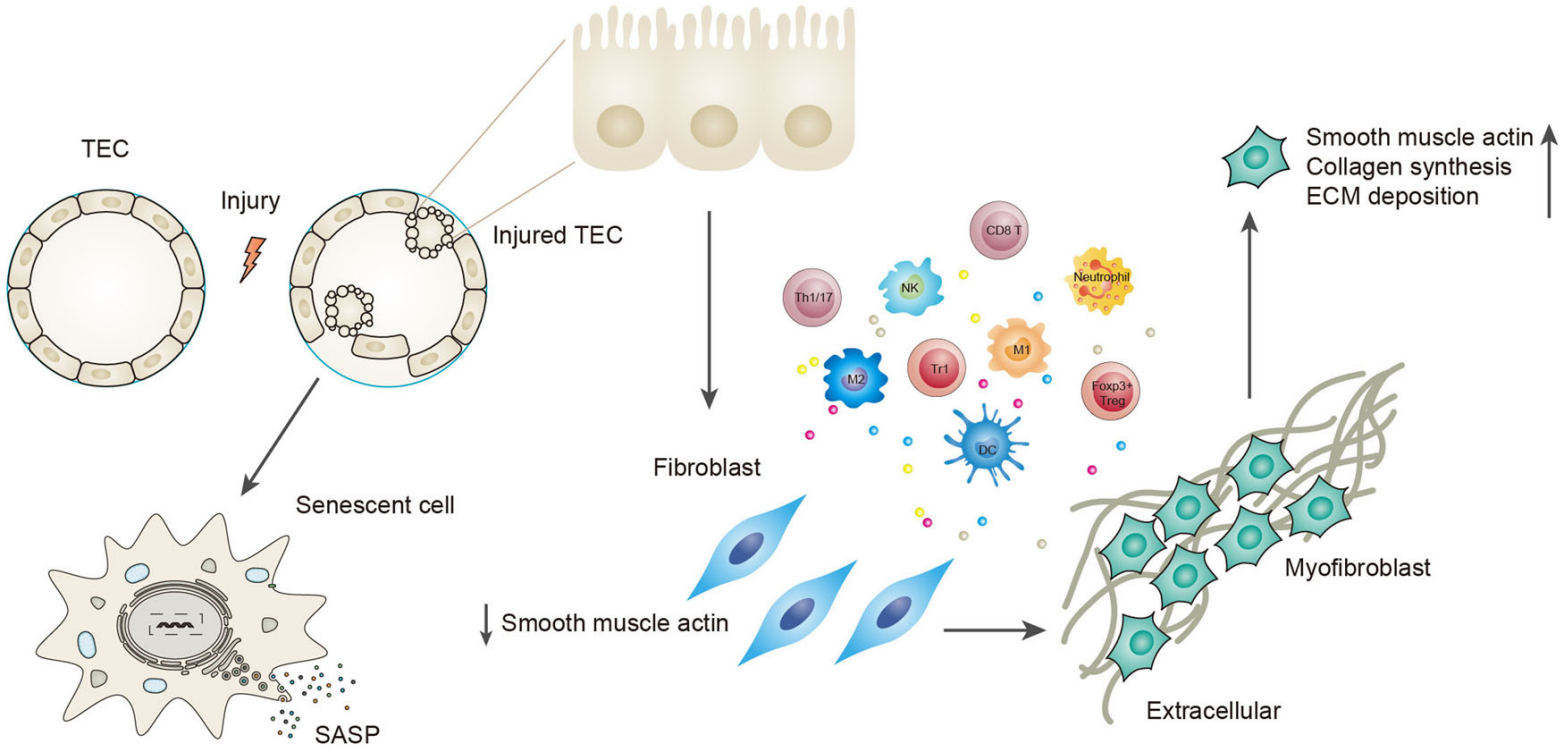
Description of maladaptive repair. It is the severity and frequency of the injury that determine whether the repair mechanism adopted by tubular cells is beneficial or maladaptive. Severe and persistent injury exceeds normal repair mechanisms, and these cells become maladaptive to survive the injury. Maladaptive repair is manifested by cell cycle arrested at the G2/M phase and a senescence-associated secretory phenotype. The former is characterized by the expression of proteins that include p53, p21, and p16^INK4a^. The latter includes secretion of proinflammatory and profibrotic factors, including TGF-beta1, CTGF, CXCL1, IL-6, IL-8, etc. Tubular cells can also undergo EMT to avoid apoptosis, with loss of some epithelial markers (E-cadherin, ZO-1) and acquisition of partial myofibroblast markers (alpha-SMA, vimentin, FSP-1). This process involves changes in the immune microenvironment. Proinflammatory factors secreted by TECs recruit and activate different inflammatory cells, and these recruited cells can further produce cytokines that drive TECs to undergo EMT. TECs finally obtain a myofibroblast phenotype, express alpha-SMA, and are responsible for collagen synthesis and ECM deposition

TECs can adopt different mechanisms upon insult and manage to regenerate and regain normal kidney function if the injury is mild and transient^67, 68^. However, if the injury is severe and persistent, normal regeneration mechanisms may not function, and TECs might undergo maladaptive repair, exacerbating renal fibrosis^67, 69, 70^ (Fig. 2). Renal fibrosis can be seen as the final consequence of improper repair mechanisms, in other words, maladaptive repair. A recent study demonstrated that it is the severity and frequency of TEC injury that determine whether the repair mechanism leads to recovery or progression to fibrosis^71^. Although adaptive repair shows profound proliferation of TECs^68^, the maladaptive response is characterized by interstitial fibrosis, tubular atrophy, and capillary rarefaction^69^.

### Cell senescence is the marker for maladaptive tubular cells

Maladaptive tubular cells manifest cell senescence^68–70^, which is defined as the absence of the ability to divide and a permanently arrested cell cycle^68, 72^. The senescence of tubular cells has been observed in CKDs induced by hypertension, diabetes, and IgA nephropathy^67^. Senescence is also observed in cell ageing. Aged mice cells have decreased cellular proliferation and increased senescence compared with young mice subjected to ischemia–reperfusion injury^73^. A proteomic study on human proximal TECs also shows increased senescence in older cells^74^. Senescence has two characteristics, cell cycle arrest at the G2/M phase and the secretion of proinflammatory and profibrotic factors;^67, 68, 70^ the latter is also known as senescence-associated secretory phenotype. These two changes are the bridges that link tubular cell senescence with renal fibrosis.

### An arrested cell cycle at the G2/M phase promotes renal fibrosis

As mentioned above, cell cycle arrest is one of the most important characteristics of cell senescence. It is the consequence of maladaptive repair by tubular cells and has a strong correlation with renal fibrosis. In uninjured kidneys, most tubular cells remain in the quiescent G0 phase. Upon insult, TECs enter the cell cycle to regenerate and replace the cells lost during injury^4^. Some cells are arrested at the G1 or G2 phase for DNA repair. However, persistent cell cycle arrest is a kind of maladaptive repair and has a strong association with the progression of renal fibrosis^67, 68, 70^. The fact that aged mice have more severe cellular senescence and fibrosis after AKI can also be explained by the increased number of cells arrested at the G2/M phase due to increased DNA damage^73^.

P53, p21, and p16^INK4a^ have been recognized as the most important factors involved in this process^5, 68, 69^. P21 is upregulated shortly after acute kidney injury (AKI). It is renoprotective in the early phase since it can halt the cell cycle to repair DNA damage, which prevents tubular cells from undergoing apoptosis^69^. The fact that *p21* knockout mice have more severe damage after AKI also demonstrates the protective role of p21 in the early-phase after injury^68^. However, persistent expression of p21 and cell cycle arrest may lead to cell senescence and aggravate fibrosis. Apart from AKI, hypoxia can also induce p21 expression and lead to G2/M phase arrest via the ataxia-telangiectasia-mutated-ataxia telangiectasia and Rad3-related R pathway^69^. P16^INK4a^ is also upregulated in tubular cells upon injury and is responsible for mediating cell cycle arrest, since deleting p16^INK4a^ could restore cell proliferation after AKI^69^. P53, the upstream regulator of p21, is also upregulated after AKI, and inhibition of p53 expression attenuates renal fibrosis^68^. P53 is also activated in DN, and its activity is in negative correlation with renal function^75^. Apart from p53, p21, and p16^INK4a^, modulation of cell cycle arrest involves more complex mechanisms. Atg5-mediated autophagy in TECs can ameliorate renal fibrosis by blocking G2/M phase arrest^37^. Macrophage migration inhibitory factor also counteracts cell cycle arrest^76^. On the other hand, miR-192 and Numb can aggravate cell cycle arrest and renal fibrosis either by upregulating or stabilizing p53^77, 78^. Cell cycle arrest is also correlated with the process of epithelial–mesenchymal transition (EMT), and overexpression of Twist1 or Snail is sufficient to prolong the G2/M arrest induced by TGF-β1^79^.Please define AKI at its first mention in text.AKI stands for acute kidney injuryPlease spell out ATM, ATR in text, as it is mentioned only once.ATM-ATR stands for ataxia-telangiectasia-mutated-ataxia telangiectasia and Rad3-related

The arrested tubular cells can express factors like TGF-β1, connective tissue growth factor (CTGF), C-X-C motif chemokine ligand 1 (CXCL1), IL-6, and IL-8, partially mediated by the JNK pathway^70^. These factors promote a chronic inflammatory microenvironment that favors fibrosis^67^. Arrested tubular cells also fail to regenerate to replace the lost cells, leaving space for the proliferation of fibroblasts and deposition of ECM. All of these changes drive the interstitium to fibrosis and aggravate renal function.Please define CXCL1 at its first mention in text.CXCL1 stands for C-X-C motif chemokine ligand 1

### EMT is a potential source of fibroblasts and ECM

EMT is referred to as the process that allows a polarized epithelial cell to assume a mesenchymal phenotype, which includes an enhanced migratory capacity, invasiveness, elevated resistance to apoptosis, and increased production of ECM components^80^. As mentioned above, TECs undergo dedifferentiation and regeneration to produce new tubular cells after injury. Meanwhile, they produce various factors that attract inflammatory cells to the tubulointerstitial space. Infiltrating cells express cytokines that further alter the tubulointerstitial microenvironment and force TECs to turn to a mesenchymal phenotype to adapt to these changes and avoid apoptosis^5, 72^. Moreover, this switch is also a potential source of fibroblasts and ECM deposition. Thus, the process of EMT becomes maladaptive and aggravates renal fibrosis.

EMT is characterized by the loss of epithelial markers, including E-cadherin and zonula occludens-1 (ZO-1), and the acquisition of mesenchymal markers, including vimentin, alpha-SMA, fibroblast-specific protein-1 (FSP-1), collagen I and fibronectin^81^. Though it is widely accepted that EMT can be induced by TGF-β1 in vitro, whether this process contributes to the production of fibroblasts in vivo is still uncertain^82^. A possible explanation for this controversy could be changes in the definition of EMT^2^. In the past, researchers expected to find complete EMT in vivo, with the complete loss of epithelial markers and the acquisition of mesenchymal markers along with tubular cells traversing the basement membrane^83, 84^. However, recent research has shown that EMT, rather than an “all or none” process, is actually a spectrum of changes^2, 4, 5^. Tubular cells could lose some epithelial makers while acquiring partial mesenchymal markers. This process is termed partial EMT, which is more likely to occur in vivo^2^.

Snail and Twist1 mediate this process^53, 85, 86^. Conditional deletion of *Snai1* or *Twist1* in TECs leads to inhibition of EMT^79^. The signaling pathway involved is complicated and overlaps with the pathways mediating renal fibrosis discussed above, including the TGF-β1/Smad pathway, intergrin/integrin linked kinase  pathway, Wnt/beta-catenin pathway, p38 MAPK pathway, Sonic hedgehog pathway, and membrane rafts–redox pathway, etc.^51, 64, 81, 87^. TECs undergoing this phenotypic switch can attract inflammatory cells and activate fibroblasts and pericytes in a paracrine manner;^4, 79^ evidence also shows that they can serve as a direct producer of ECM that can aggravate TIF^88^.Please spell out ILK in text, as it is mentioned only once.ILK stands for integrin linked kinase

EMT and cell senescence, manifested by G2/M phase arrest, actually have much in common. Both occur after persistent injury and maladaptive repair; both go through the process of dedifferentiation, and the secretion of proinflammatory and profibrotic cytokines, which aggravates renal fibrosis, is seen in both. Though not confirmed by experiments, it may be reasonable to infer that they are both the consequences of maladaptive repair. Moreover, they may even be two inseparable facets of the same process, with EMT emphasizing phenotypic changes and senescence emphasizing cell cycle changes. However, more research is needed to prove a correlation between EMT and cell senescence and to provide new insights into therapeutic approaches for targeting maladaptive repair mechanisms.

Though most of the researches discussed above were conducted in native kidneys, the unique nature of kidney transplantation, for example, the ischemia injury during harvesting the kidney, immune-mediated injury, use of immunosuppressants, and sequential protocol biopsies make allograft an ideal target studying the correlation between TECs and renal fibrosis. The term “interstitial fibrosis/tubular atrophy (IF/TA)” is often used in transplant biopsies, given that these two phenomena almost inevitably occur in parallel^89, 90^. Most of the underlying mechanisms discussed above are also confirmed in allograft studies. Take EMT for example, extensive biopsy studies have demonstrated the mesenchymal phenotype change of tubular cells in vivo, which contributes to the progression of fibrosis in the allograft and is predictive for graft loss^91–93^. Growing numbers of studies are focusing on counteracting TIF to extend graft survival time. Mesenchymal stem cells have been confirmed to play a renoprotective role in transplanted kidneys by attenuating tubular atrophy and TIF^94–96^. Given the unique characteristics, renal allograft might be a promising model studying renal fibrosis in the future.

## How do TECs interact with other cell types in the interstitium to advance renal fibrosis?

**Fig. 3:**
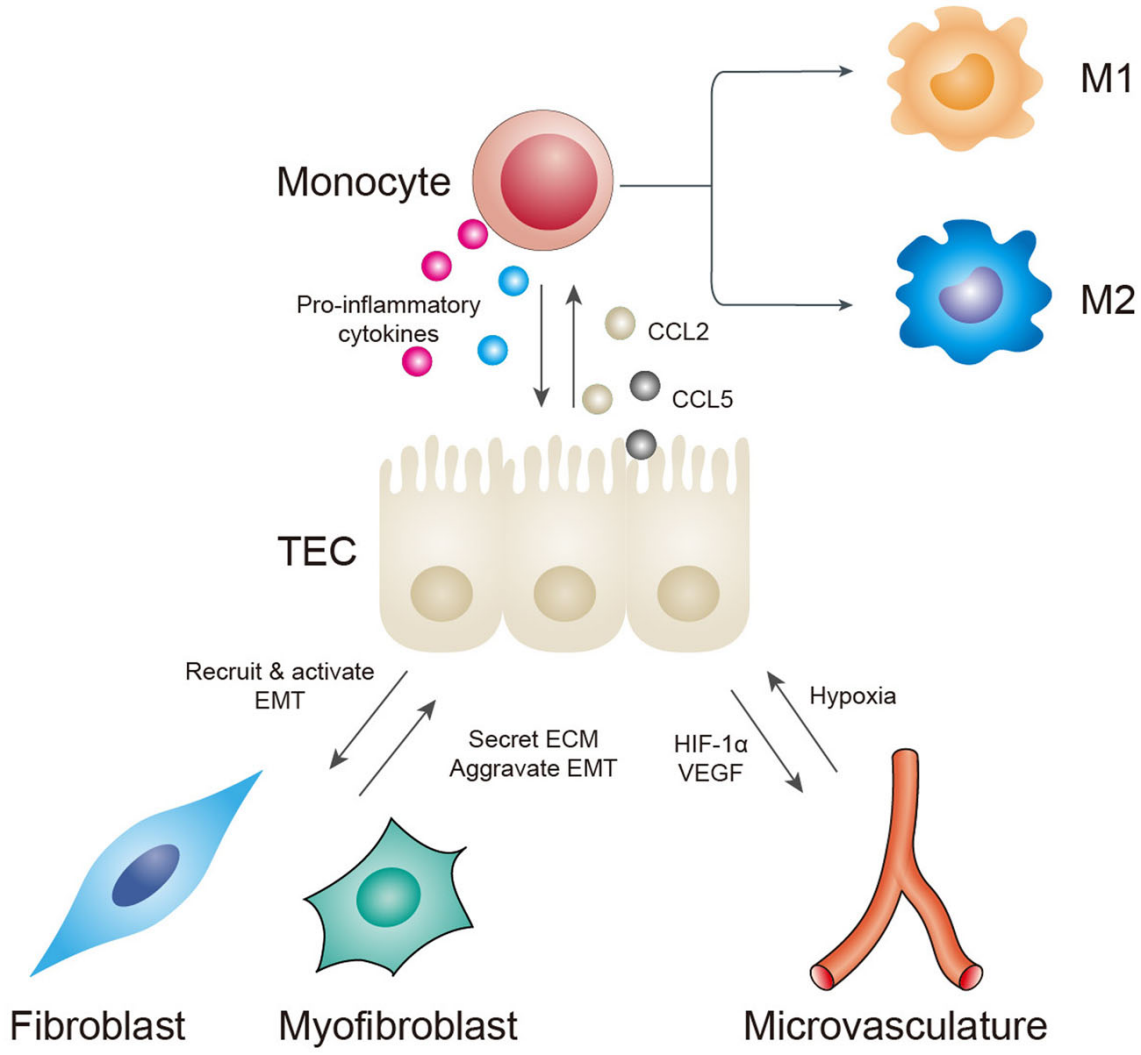
Interactions between TECs and other cells in the interstitium. TECs can secrete CCL2 and CCL5 to recruit monocytes. Monocytes/macrophages can further produce proinflammatory factors, but their functions depend on their polarization phenotypes. Monocytes can also induce EMT by producing MMP-9. Capillary rarefaction causes hypoxia in tubular cells. Injured TECs thus secrete HIF-1alpha and VEGF to stimulate new capillary formation to meet their oxygen demand. However, these new capillaries are often leaky and are incapable of performing normal functions, thus forming a vicious cycle. Maladaptive TECs can produce a microenvironment suitable for fibroblast recruitment and activation. Activated myofibroblasts can mediate ECM deposition and execute the final process of renal fibrosis. Increased matrix rigidity can also aggravate tubular hypoxia and the progression of EMT

Growing evidence has shown that TECs are indispensable mediators in TIF; they must interact with other cells in the interstitium, including inflammatory cells, endothelial cells, fibroblasts, and pericytes, to drive the progression of fibrosis. Maladaptive TECs can either attract or activate these cells via crosstalk, and these interactions together cause the progression of TIF (Fig. 3).

### Crosstalk between TECs and inflammatory cells

Inflammation has been widely observed in a variety of CKD biopsy specimens and animal models and has been recognized as crucial for the initiation of renal fibrogenesis^2^. Among the inflammatory cells, the role of monocytes/macrophages is the most well-established.

Maladaptive TECs can secrete CCL2 and CCL5 to recruit monocytes and macrophages^97–99^. Peripheral monocytes that are recruited can differentiate into macrophages once they arrive at the renal interstitium^98^. Interestingly, a recent study demonstrates that recruited monocytes could instruct TECs to turn off chemokine secretion and downregulate the production of proinflammatory factors;^98^ this self-limited negative feedback system indicates that monocyte may also play an important role in the resolution of inflammation. TECs can also recruit macrophages by secreting matrix metallopeptidase 9 (MMP-9) in the early stage after UUO, whereas recruited macrophages produce more MMP-9, which can aggravate the progression of EMT^100^.Please define MMP-9 at its first mention in text.MMP-9 stands for matrix metallopeptidase 9

Once recruited, macrophages exert pleiotropic effects on renal fibrosis depending on the polarization form: a proinflammatory M1 phenotype or a reparative M2 phenotype^2^. It has been well documented that, shortly after AKI, predominant M1 macrophages can amplify damage, whereas the later M2 phenotype is responsible for repair^67, 99^. However, persistent appearance of M2 macrophages leads to improper repair and is strongly correlated with fibrosis in CKD^99^. Transformation of the macrophage phenotype is determined by the microenvironment in the interstitium.

Activated macrophages produce a variety of molecules, including tumor necrosis factor-alpha, platelet-derived growth factor (PDGF), basic fibroblast growth factor (FGF-2), TGF-β, and ROS, which induce further inflammation and fibrosis^101^. Soluble uric acid can stimulate the NLRP3 inflammasome/IL-1β pathway in macrophages and in turn activates cytokine production in tubular cells^102^. Again, the effects of macrophages on tubular cells and renal fibrosis depend on the polarization phenotype, as well as the stages after injury.Is the insertion of ‘tumor necrosis factor’ as the definition of ‘TNF’ correct?Yes, TNF stands for tumor necrosis factor.

The effects of other inflammatory cells on fibrogenesis are less clear. It has been shown that T cells may play an initiation role in inflammation, as they precede the influx of macrophages into the kidney after injury^2^. High glucose-induced macrophage inflammatory protein-3 alpha from TECs is responsible for recruiting CD3^+^ cells to the interstitium^103^. A recent study has shed new insight on the roles of different subpopulations of T cells^104^. Researchers found that the depletion of either CD4^+^ or CD8^+^ T cells leads to aggravated renal fibrosis^104^. Accumulation of B cells in the kidney can promote monocyte/macrophage recruitment by the secretion of CCL2, thus aggravating renal fibrosis. NK cells and C3aR^+^ cells are also positively correlated with TIF, but the mechanisms involved remain unclear^105, 106^.

The complement is also activated during renal fibrosis^107^. An increased C5a level is observed in tubular cells in DN and is positively correlated with disease progression^108^. Local synthesis of the complement is also observed in PDGFRbeta-positive pericytes and CD45 (+) cells in the interstitium^109^. Thus, blocking synthesis or activation of the complement at different levels may offer benefits for renal fibrosis^107^. Tubular cells may also be part of the innate immune response. Released damage-associated molecular patterns can activate TECs by binding to their pattern recognition receptor, such as TLRs. Activated TECs can then produce cytokines or undergo pyroptosis, thus forming an auto-amplification loop of tubulointerstitial inflammation^5^.

### Crosstalk between TECs and the microvasculature

Intact microvasculature is a prerequisite for normal tubular structure and function. Peritubular capillaries and tubular cells are often injured together during renal injuries. Loss of peritubular capillaries is strongly correlated with renal fibrosis. A decrease in CD34^+^ tubulointerstitial capillaries is observed together with TIF in the UUO model^73, 110^. However, it is hard to determine whether pericapillary injury is the cause or consequence of renal fibrosis. Capillary rarefaction results in hypoxia in tubular cells, thereby increasing oxidative stress in the interstitium. This, in turn, aggravates the damage in the microvasculature, forming a vicious cycle^75^. Tubular cells demand oxygen to fulfill their normal functions. Hypoxia-inducible factor-1alpha (HIF-1alpha) and its downstream vascular endothelial growth factor (VEGF) are upregulated in TECs during hypoxia^111^. VEGF plays a dual role in the microvasculature. As a pro-angiogenic factor, it promotes endothelial cell survival and proliferation, thus enhancing peritubular capillary density^112^. However, excessive VEGF may result in the formation of leaky and nonfunctional vessels, which further aggravates hypoxia and oxidative stress in the interstitium^99^. Additionally, it is reported that VEGF can act as a proinflammatory factor that exacerbates renal fibrosis^99^. Secretion of anti-angiogenic factors, such as endostatin and thrombospodin-1, by TECs can lead to microvasculature damage^72^, and this demonstrates the protective role of VEGF. Angiopoietin-1, a secreted vascular growth factor, ameliorates capillary rarefaction when overexpressed in TECs^113^.Please define HIF-1alpha at its first mention in text.HIF-1alpha stands for Hypoxia-inducible factor-1alpha

The close contact of pericytes with the endothelium is also important in maintaining capillary stability. A recent study demonstrates that ablation of pericytes alone could lead to endothelial damage and capillary rarefaction and further causes subclinical tubular cell injury, which is detected by expression of kidney injured molecule-1 and vimentin in TECs^114^. It is also reported that pericytes are a potential source of myofibroblasts. Pericytes can undergo a phenotype switch similar to EMT and acquire myofibroblast characteristics upon injury^115^. Thus, detachment of pericytes from the capillaries may further aggravate capillary rarefaction by forming more myofibroblasts.

### Crosstalk between TECs and fibroblasts/myofibroblasts

Fibroblasts and myofibroblasts are the final executors of renal fibrosis. Upon injury, maladaptive TECs give rise to a switch in the microenvironment in the tubulointerstitial space in which myofibroblasts are activated. These activated executors are capable of producing substantial ECM, leading to TIF. TECs and myofibroblasts are at the two ends of the progression of renal fibrosis, the former as the initial reactor after injury and the latter as the final executor. Thus, understanding the crosstalk between these two cell types is of great importance.

Myofibroblasts are the activated form of fibroblasts, and they are characterized by the expression of alpha-smooth muscle actin (alpha-SMA), proliferation, and matrix production^2^. Recent studies have suggested five major origins of myofibroblasts, including resident fibroblasts, TECs, endothelial cells, pericytes, and bone marrow-derived fibrocytes^2, 4, 115^. However, the exact contribution of each origin to renal fibrosis remains unclear. Tsutomu et al. reviewed several lineage-tracing experiments and found conflicting results regarding the contribution of EMT to renal fibrosis^82^. They proposed that the controversial results might be attributed to different disease models, mouse strains and types of genetic alterations used. They further demonstrated this hypothesis with four different models and confirmed that EMT-derived fibroblasts are activated in a disease-specific and strain-dependent manner^82^. In other words, in different conditions, the five origins of fibroblasts account for different proportions of intrarenal myofibroblasts. The different results could also be accounted for the inherent heterogeneity of myofibroblasts^2, 115^. The heterogeneity of myofibroblasts also leads to the lack of a specific marker to identify myofibroblasts^2^.

Despite the conflicting origins, it has been a common view that myofibroblasts are mainly activated by a variety of cytokines and growth factors produced by TECs in a paracrine manner, including TGF-beta1, Wnt ligands, PDGF, hedgehog, hepatocyte growth factor, CTGF, and angiotensin II^99, 116–118^. Recent studies have shed light on some new factors produced by TECs, such as FGF-23. According to a recent study, FGF-23 could activate myofibroblasts derived from obstructed kidneys but fail to activate fibroblasts derived from normal kidneys^119^. FGF-23 could also induce ECM production in rat renal TECs (NRK-52E) via the TGF-β1 pathway^120^. Another study using immunofluorescence analysis also confirmed that TECs can act as an efficient producer of stable ECM^88^. The EGFR pathway is also involved in the TEC-fibroblast crosstalk. Persistent epithelial-specific activation of EGFR leads to epithelial dedifferentiation and cell cycle arrest, along with promoting proliferation of alpha-SMA^+^ myofibroblasts^59^.

On the other hand, activated myofibroblasts can also exert profibrotic effects on tubular cells. Accumulation of ECM results in increased matrix rigidity and other biomechanical changes in the interstitium, which in turn leads to augmented levels of TGF-β1 expression and EMT^121^. ECM can also increase the diffusion distance of oxygen, thereby worsening hypoxia in TECs^4^. Microvesicles derived from fibroblasts can deliver miR-34a to tubular cells and induce tubular cell apoptosis^122^.

## Final considerations

Growing numbers of studies have demonstrated that the long-neglected TECs are important mediators of TIF after injury. TECs can adopt different repair mechanisms to survive injury and recover normal kidney function. However, long-lasting stress makes these mechanisms maladaptive. The completion of fibrosis depends on crosstalk between TECs and other cells in the interstitium. Understanding the importance of TECs can help us further comprehend the complicated mechanisms of renal fibrosis. In the future, TECs may become new therapeutic targets to ameliorate or even reverse this process. Further investigations are needed in this field to explore the elaborate changes in TECs upon injury and how they interact with other cells to mediate renal fibrosis.
